# Supplementary material for: Regulation of coronavirus nsp15 cleavage specificity by RNA structure
Source: PLoS One. 2023 Aug 24;18(8):e0290675. doi: 10.1371/journal.pone.0290675 (PMC10449227; doi:10.1371/journal.pone.0290675)

Uncropped gel for Fig 1C. Gel was captured with BioRad GelDock+ using the SYBR Green protocol.

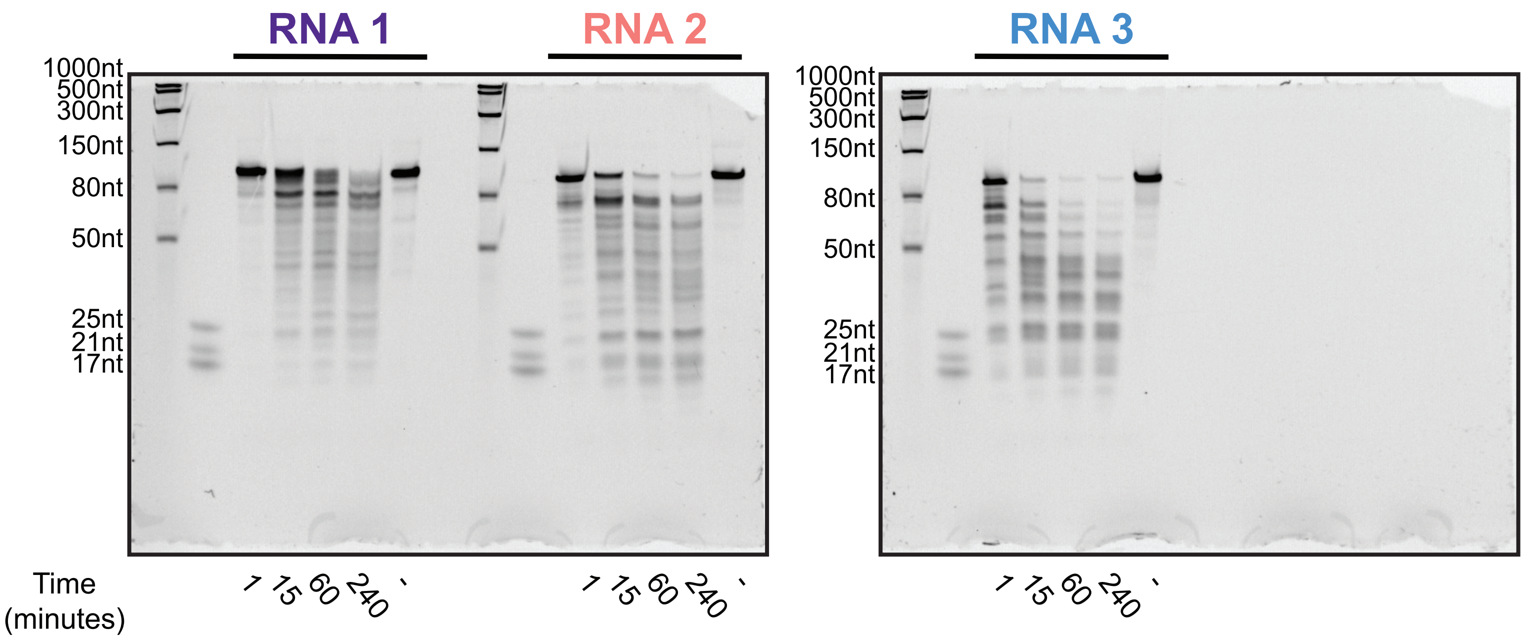

Uncropped gel for Fig 2A. Gel was captured with BioRad GelDock+ using the SYBR Green protocol.

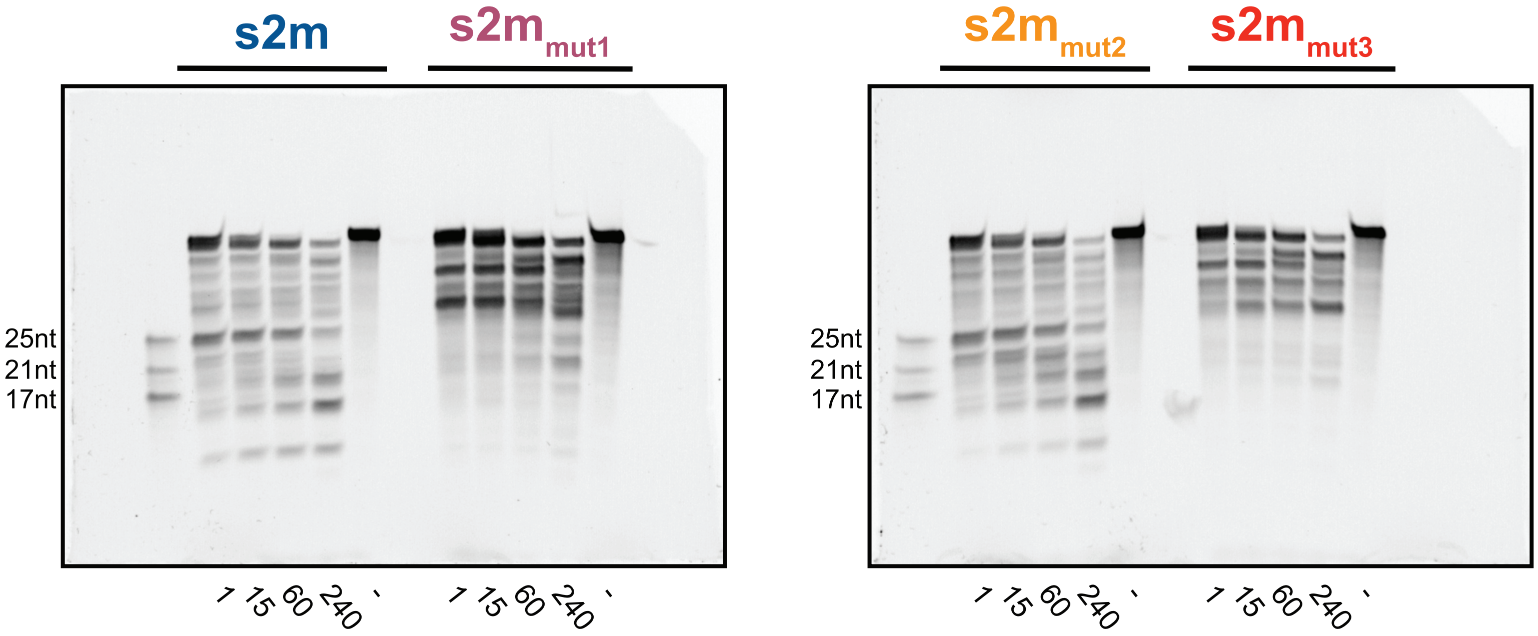

Uncropped gel for Fig 2D. Gel was captured with BioRad GelDock+ using the SYBR Green protocol.

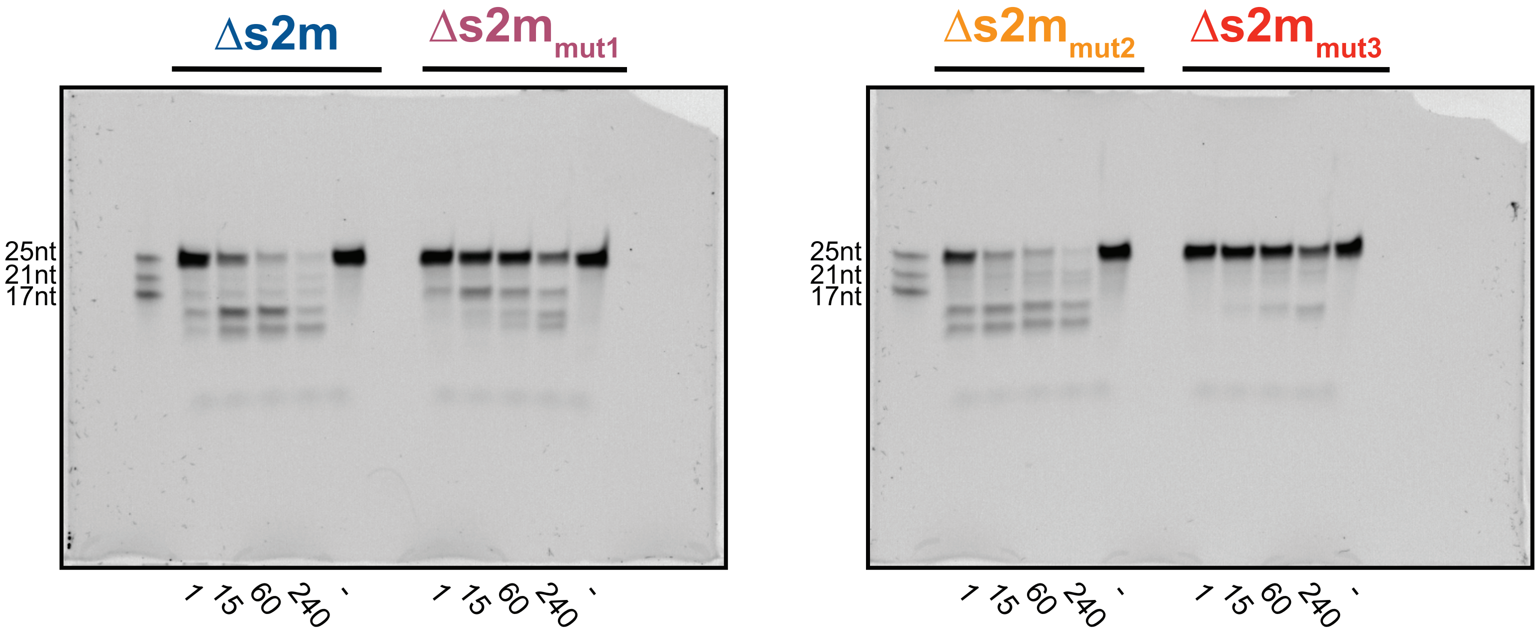

Uncropped gel for Fig 3A. Gel was captured with BioRad GelDock+ using the SYBR Green protocol.

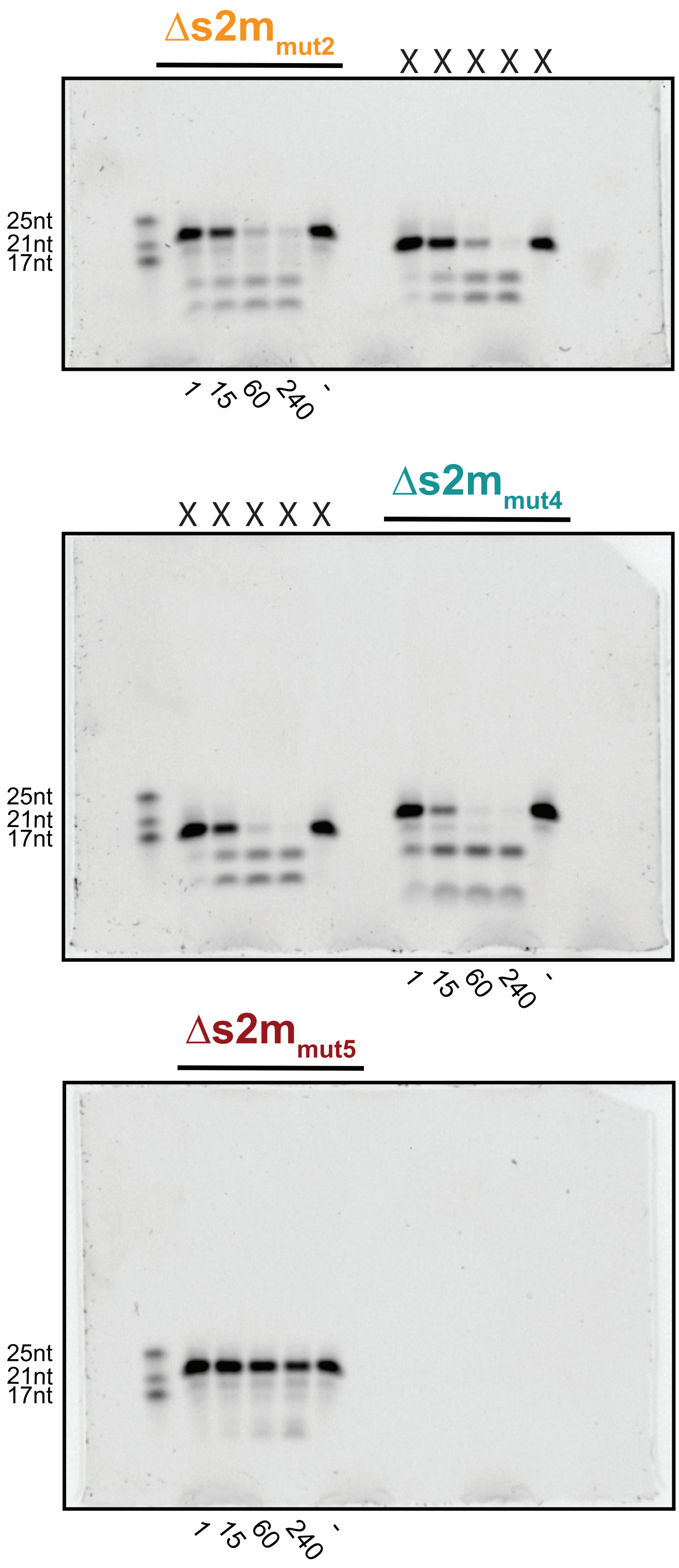

Uncropped gel for S1v Fig. Gel was captured with BioRad GelDock+ using the SYBR Green protocol.

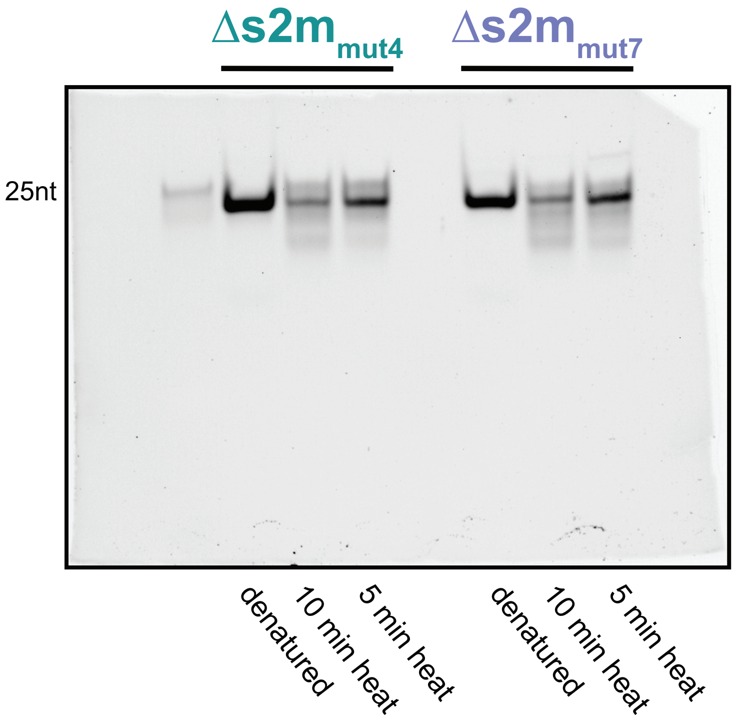

Uncropped gel for S2 Fig. Gel was captured with BioRad GelDock+ using the SYBR Green protocol.

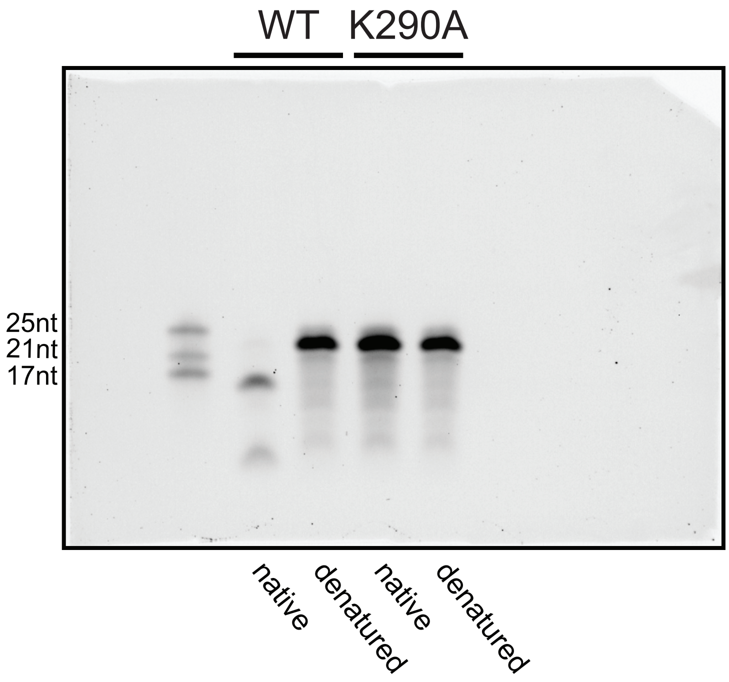

Uncropped gel for S6A Fig. Gel was captured with BioRad GelDock+ using the SYBR Green protocol.

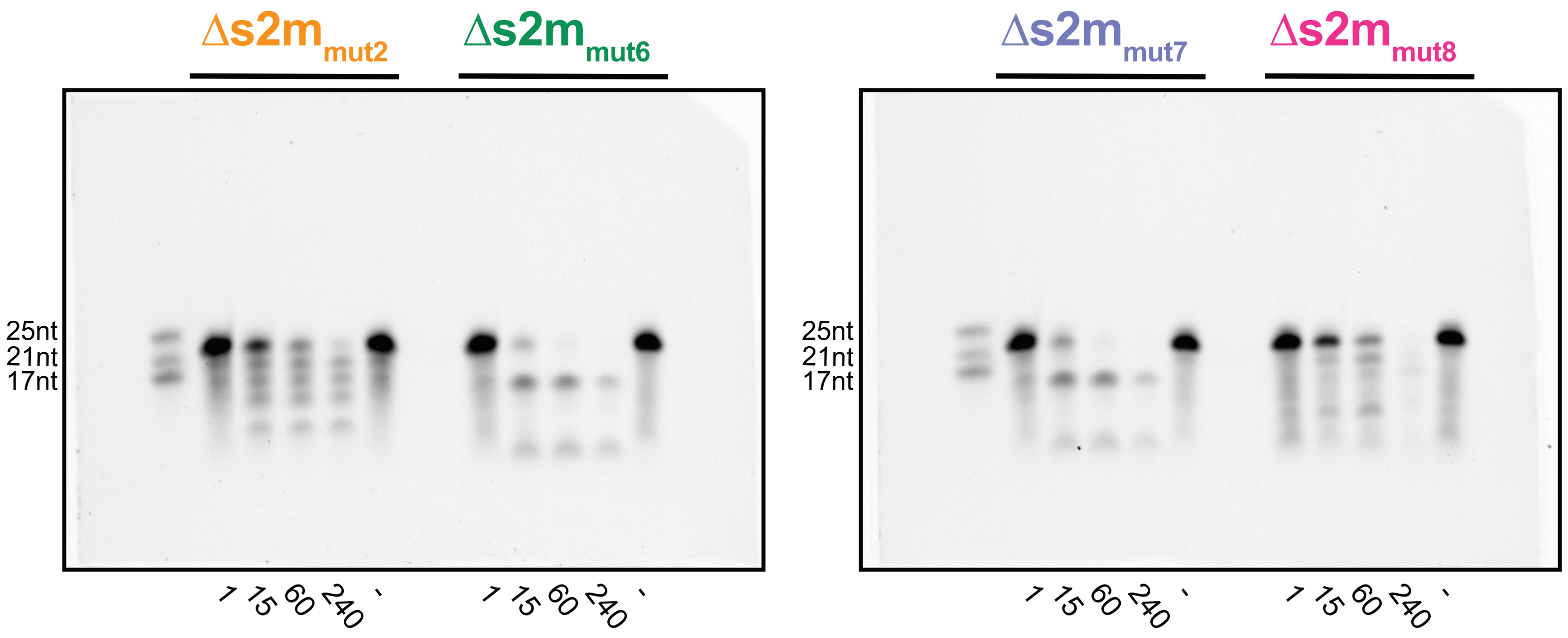

Uncropped gel for S7A Fig. Gel was captured with BioRad GelDock+ using the SYBR Green protocol.

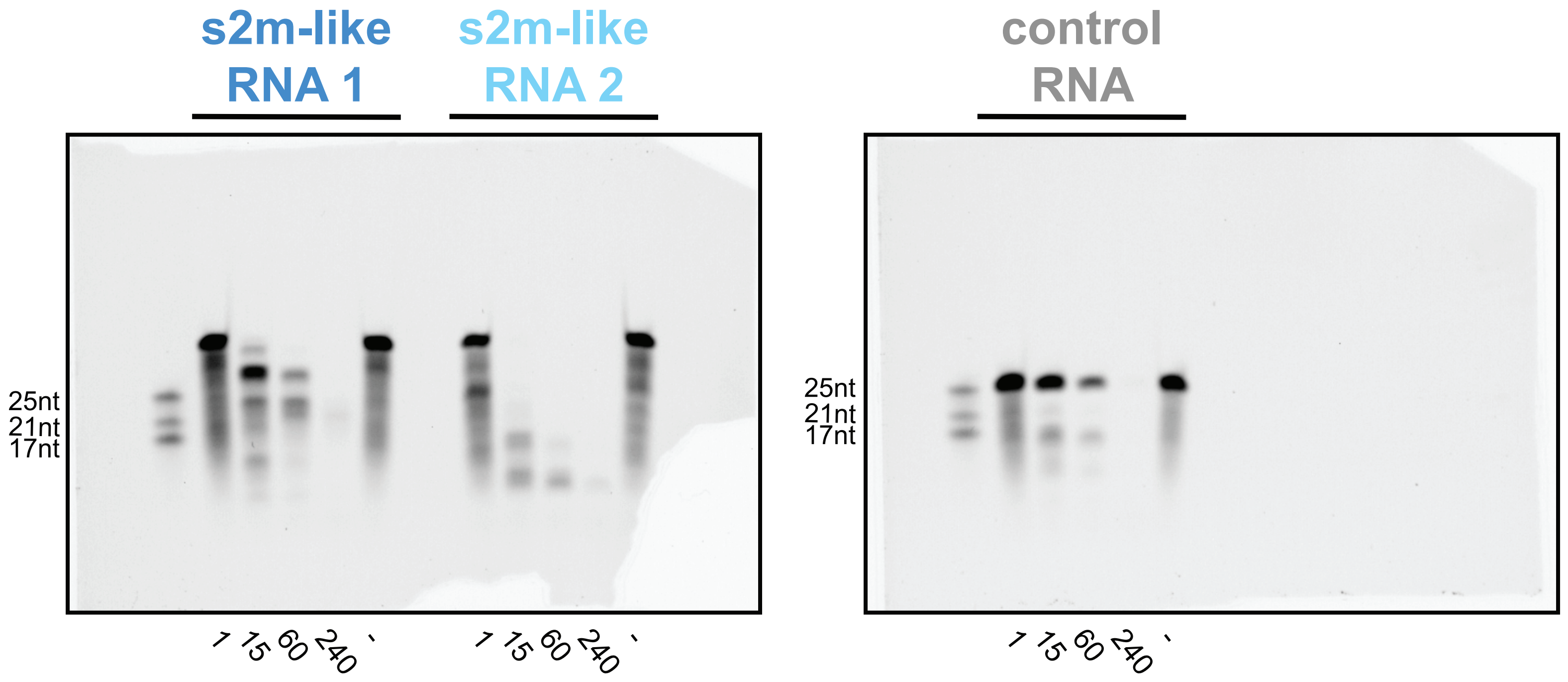

Uncropped gel for S8 Fig. Gel was captured with BioRad GelDock+ using the SYBR Green protocol.

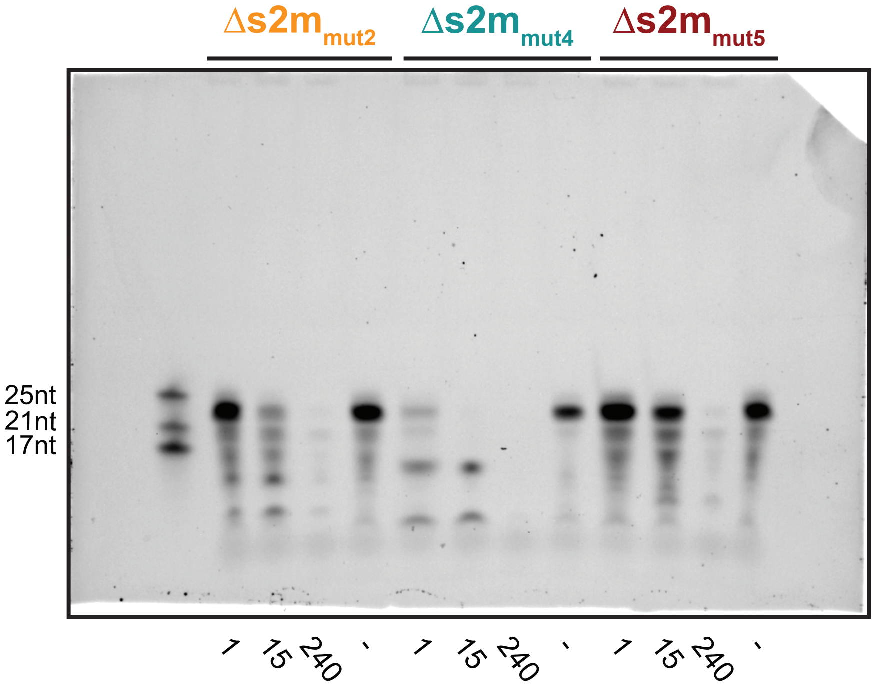

Supplement: S1 Raw images — (PDF) [file pone.0290675.s010.pdf]
